# Acupuncture Improves Functional Limitations for Cancer Patients with Chronic Pain: A Secondary Analysis of PEACE Randomized Clinical Trial

**DOI:** 10.3390/curroncol32110640

**Published:** 2025-11-16

**Authors:** Lingyun Sun, Mothi Babu Ramalingam, Raymond Baser, Marco Santos Teles, Christina Seluzicki, Qing Susan Li, Jun J. Mao

**Affiliations:** 1 Oncology Department, Xiyuan Hospital, China Academy of Chinese Medical Sciences, Beijing 100091, China; 2 Integrative Medicine Service, Department of Medicine, Memorial Sloan Kettering Cancer Center, New York, NY 10065, USA; 3 Department of Rehabilitation Medicine, Singapore General Hospital, Singapore 169856, Singapore; 4 Epidemiology-Biostatistics, Memorial Sloan Kettering Cancer Center, New York, NY 60637, USA

**Keywords:** cancer pain, acupuncture, rehabilitation, extremity function, randomized controlled trial

## Abstract

Chronic pain significantly impairs functional performance in patients with cancer. Although acupuncture is effective for cancer-related pain, its impact on pain-related functional interference remains unclear. This secondary analysis of the PEACE randomized clinical trial included patients with prior cancer diagnoses and musculoskeletal pain for ≥3 months. Participants were randomized to groups undergoing 10 weeks of electro-acupuncture, auricular acupuncture, or a waitlist control. Functional performance was assessed using the Quick-Disability Arm/Shoulder/Hand (Q-DASH) for upper limbs and the Western Ontario and McMaster Universities Osteoarthritis (WOMAC) subscale for lower limbs (higher scores = worse function). Linear mixed models compared changes over time between groups, with week 12 as the primary endpoint. Functional changes were also compared between pain responders and non-responders in the acupuncture arms. Among 360 patients (mean [SD] age, 62.1 [12.7] years; 69.7% women), mean baseline Q-DASH and WOMAC scores were 33.2 (19.8) and 33.3 (20.3). At week 12, both electro-acupuncture and auricular acupuncture significantly improved function versus waitlist: Q-DASH by −7.18 and −9.64 points, respectively, and WOMAC by −6.89 and −7.61 points (all *p* < 0.001). No differences were found between the two acupuncture groups. Treatment effects on Q-DASH diminished during follow-up, while improvements on WOMAC persisted. Within the acupuncture groups, pain responders achieved greater functional gains than non-responders (Q-DASH, −6.74; WOMAC, −6.16; both *p* < 0.001). Electro-acupuncture and auricular acupuncture improved upper and lower extremity function in cancer patients with chronic pain. These findings support acupuncture as a potential adjunct in functional rehabilitation for cancer survivors.

## Simple Summary

This study investigated the effects of electro-acupuncture and auricular acupuncture on pain-related functional impairment in cancer patients with chronic musculoskeletal pain. As a secondary analysis of a randomized clinical trial, participants received acupuncture or were placed on a waitlist control. Functional outcomes for upper and lower limbs were measured using standardized scales. Both forms of acupuncture improved functional performance compared with the control group, with benefits varying over time between upper and lower extremities. Additionally, patients who experienced pain relief showed greater functional improvement. These findings suggest that acupuncture may serve as an effective adjunct therapy to enhance physical function and rehabilitation among cancer survivors with chronic pain.

## 1. Introduction

Chronic pain is very common among patients with cancer and is strongly associated with impaired physical functioning [1,2]. A large populational study showed that 54.7% of patients with cancer with high-impact pain reported severe physical functioning limitations [3]. Such limitations on body and extremity function restrict patients’ mobility to attend to daily activities and exercise, leading to reduced overall quality of life [4,5,6]. Studies have shown that pain-related functioning limitation is related to worse cancer treatment responses and even survival outcomes [7,8,9,10]. Current interventions for functional limitations include physical therapy, exercise, and myofascial therapy [11]. However, real-world studies have shown that pain itself could impair patients’ compliance with these interventions [12]. According to national data, patients with severe pain and more comorbidities were more likely to no-show for their scheduled physical therapy session [13]. Another study found that among patients who did not adhere to an exercise-based physical therapy program, those with worse baseline pain were more likely to withdraw [14]. Therefore, identifying interventions that can reduce pain and improve functions may be important for patients’ rehabilitation.

Acupuncture is an effective nonpharmacologic intervention for managing cancer-related pain and has been incorporated into clinical guidelines for pain management [15]. In the general population, acupuncture has been shown to enhance functional performance in patients with chronic pain conditions, including non-specific lower back pain, neck pain, and osteoarthritis [16,17,18]. However, functional limitations due to cancer pain may differ, as patients with cancer often experience additional physical and psychological symptom burden [19,20]. Nevertheless, there remains a critical gap in evidence for acupuncture’s effects on functional performance in patients with cancer-related pain. Understanding such evidence is critical to help patients with cancer not only obtain relief from pain but also recover functional performance to improve their quality of life, allowing for more holistic and patient-centered care.

Electro-acupuncture and auricular acupuncture represent two commonly applied modalities for managing cancer-related pain. In our prior Personalized Electro-acupuncture versus Auricular Acupuncture Comparative Effectiveness (PEACE) trial, we demonstrated that both approaches effectively alleviated pain intensity in cancer patients compared with a waitlist control group [21]. In this secondary analysis of the PEACE trial, we aimed to evaluate the effect of both electro-acupuncture and auricular acupuncture on the functional performance of extremities and the durability of treatment effects. We further aimed to investigate the association between pain relief and functional improvement in patients receiving acupuncture. We hypothesized that both electro-acupuncture and auricular acupuncture could improve upper and lower extremity function for patients with cancer experiencing pain when compared with a waitlist control, and that such improvement is associated with patients’ treatment response to pain.

## 2. Methods

### 2.1. Study Design

The current secondary analysis utilized data from the PEACE trial (ClinicalTrials.gov Identifier: NCT02979574), a multicenter randomized controlled study that included three groups: electro-acupuncture, auricular acupuncture, and a waitlist control. Individuals with cancer-related chronic musculoskeletal pain were randomized in a 2:2:1 ratio to receive ten sessions of either electro-acupuncture or auricular acupuncture, or to continue with usual pain management for ten weeks. Randomization procedures were stratified by study location and patients’ baseline opioid use. The main outcome of the initial randomized trial was the change in pain intensity, measured by the Brief Pain Inventory (BPI) from baseline through week 12. The study took place from March 2017 to October 2019 and was approved by the Institutional Review Board of Memorial Sloan Kettering Cancer Center (IRB No. 16-1579 A (15)).

### 2.2. Study Population

The original RCT enrolled patients with any type of cancer diagnosis, having musculoskeletal pain with a BPI score higher than 4 that lasted for at least 3 months. In the current secondary analysis, we included patients from the ITT dataset who had provided functional assessment data.

### 2.3. Intervention

The intervention of the RCT had been published previously [21].

Elctro-acupunctureCertified acupuncturists administered ten sessions over a ten-week period using a semi-standardized treatment protocol. Electrical stimulation (2 Hz) was applied with an A3922 E-STIM II device (Tens Plus Industrial Company, Kowloon, Hong Kong, China) to four acupoints located near the site of pain. Each treatment lasted 30 min, after which all needles were removed.

Auricular Acupuncture: The same team of acupuncturists performed auricular acupuncture following the U.S. military’s battlefield acupuncture protocol. After disinfecting the auricular surface, up to ten needles were inserted at predetermined auricular points, selected according to each patient’s pain response. Each session lasted approximately 10–20 min, depending on the number of needles used. Needles remained in situ for three to four days, and participants completed ten sessions across ten weeks.

Waitlist Control: Participants assigned to the control group continued to receive routine pain management as directed by their healthcare providers, which could include analgesic medication, physical therapy, or corticosteroid injections. After completing the 12-week observation period, patients were given the option to receive ten sessions of acupuncture.

### 2.4. Outcomes

In this secondary analysis, the primary outcomes were the changes in extremity functional performance measured by Quick-Disability Arm/Shoulder/Hand (Q-DASH) and Western Ontario and McMaster Universities Osteoarthritis (WOMAC) Physical Function subscale from baseline to week 12. Q-DASH is an 11-item questionnaire that measures disability related to arm, shoulder, and hand symptoms [22]. Each item has 5 response options, and, from the item scores, a Q-DASH score is calculated, ranging from 0 (no disability) to 100 (most severe disability). A higher score indicates greater disability in the upper extremities. The WOMAC physical function subscale has 17 questions, which have excellent and construct validity to measure physical function in people with hip or knee osteoarthritis [23]. For each question, patients choose the degree of functional difficulty experienced, ranging from “0—none,” “1—mild,” “2—moderate,” “3—severe,” to “4—extreme.” The WOMAC physical function score is calculated by summing the item scores and then transforming the sums to range from 0 to 100; higher scores indicate greater functional difficulty in the lower extremities that the patient is experiencing. Participants completed study assessments at baseline (week 0) and at weeks 4, 10, 12, 16, and 24 using the Research Electronic Data Capture (REDCap) system.

### 2.5. Statistical Analysis

Demographic and baseline clinical characteristics were summarized with descriptive statistics. Continuous variables (including age, baseline Q-DASH and WOMAC scores, baseline BPI pain severity, time since cancer diagnosis, and pain duration) were described using means and standard deviations (SDs). Categorical variables (such as sex, race, cancer type, cancer treatment, and pain medication use) were summarized as frequencies and percentages. For function performance outcomes, our primary hypothesis was that electro-acupuncture or auricular acupuncture compared to a waitlist control would lead to significant improvements in upper and lower extremity function measured by the Q-DASH and WOMAC at 12 weeks. Changes in Q-DASH and WOMAC scores over time were analyzed using a constrained linear mixed-effects model. This model assumed a common baseline mean across treatment groups, consistent with the pre-randomization timing of baseline assessments. Fixed effects included treatment arm, assessment time, their interaction, and the stratification factors (study site and baseline opioid use), while patient-level random intercepts were incorporated to account for within-subject correlation. All randomized patients with at least 1 outcome assessment were included in the model. For the WLC group, only data within 12 weeks of the study phase are presented.

Among the two acupuncture arms (electro-acupuncture and auricular acupuncture), we compared Q-DASH and WOMAC scores over time by pain response. Following established BPI-based criteria for analgesic response, participants achieving a ≥30% reduction in BPI pain severity from baseline to week 10 were classified as pain responders [24]. Then, we compared Q-DASH and WOMAC-function scores over time by BPI response (pain responder or non-responder) using linear mixed models with BPI response, assessment time, and the BPI response-by-time interaction as fixed effects, controlling for study site, baseline opioid use, and treatment arm. The models also included patient-specific random intercepts. A two-sided *p* value of <0.05 was regarded as statistically significant. The sample size of the parent study was determined to provide 80% power to detect the primary endpoint while maintaining a 5% overall Type I error rate. All statistical analyses were conducted using R software (version 4.4.3) [25].

## 3. Results

### 3.1. Patient Information

Baseline demographic and clinical characteristics were balanced across the three study arms (Table 1). The mean (SD) age of participants was 62.1 (12.7) years; 251 (69.7%) were women, and 88 (24.4%) were non-White. Mean (SD) baseline functional scores were 33.2 (19.8) for the Q-DASH and 33.3 (20.3) for the WOMAC-function subscale, indicating moderate impairment. No significant between-group differences were observed at baseline.

### 3.2. Q-DASH

**Figure 1:**
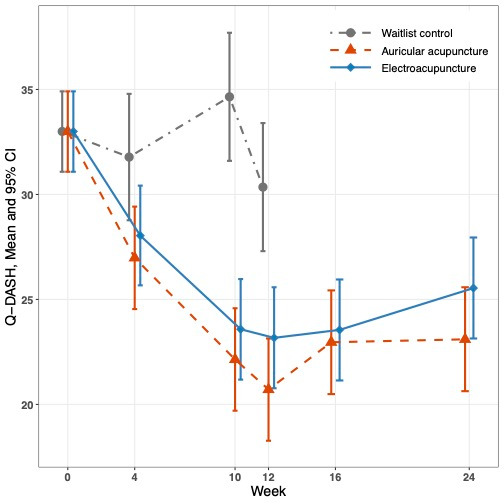

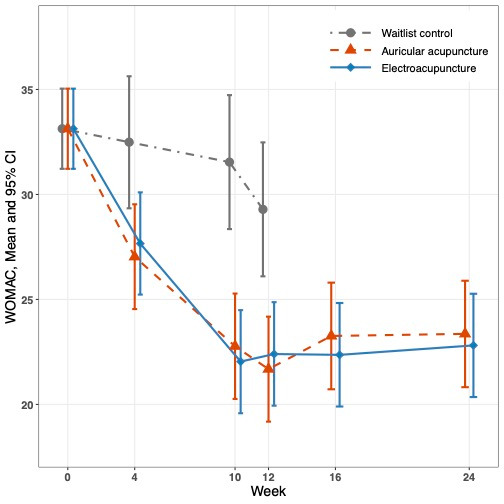
Q-DASH and WOMAC scores between treatment groups over 24 weeks.

Compared with waitlist control, the reduction in Q-DASH score was 7.18 points (95% confidence interval [CI] 3.96, 10.39, *p* < 0.001) greater with electro-acupuncture and was 9.64 points (95% CI 6.40, 12.89, *p* < 0.001) greater from baseline to week 12 (Figure 1 and Table 2). The reduction in Q-DASH score was 2.47 points greater with auricular acupuncture than with electro-acupuncture, but the difference was not statistically significant (*p* = 0.068). Compared to baseline, both acupuncture groups still had significantly lower Q-DASH scores at week 24. However, compared to week 12, the week 24 scores in the electro-acupuncture groups were 2.37 (95% CI 0.40, 4.33) points higher (*p* = 0.018), and in the auricular acupuncture group, they were 2.40 (95% CI 0.36, 4.43) points higher (*p* = 0.021).

### 3.3. WOMAC

Compared with waitlist control, electro-acupuncture had a reduction in WOMAC score that was 6.89 points (95% CI 3.44, 10.33, *p* < 0.001) greater and auricular acupuncture patients had a reduction that was significantly reduced average WOMAC score by 7.61 points (95% CI 4.14, 11.08, *p* < 0.001) greater than waitlist control from baseline to week 12 (Figure 1 and Table 2). There was no significant difference in WOMAC score between auricular acupuncture and electro-acupuncture (*p* = 0.61). In both acupuncture groups, the reduction in average WOMAC-function persisted until week 24.

### 3.4. Pain Response and Extremity Function Improvement

**Figure 2:**
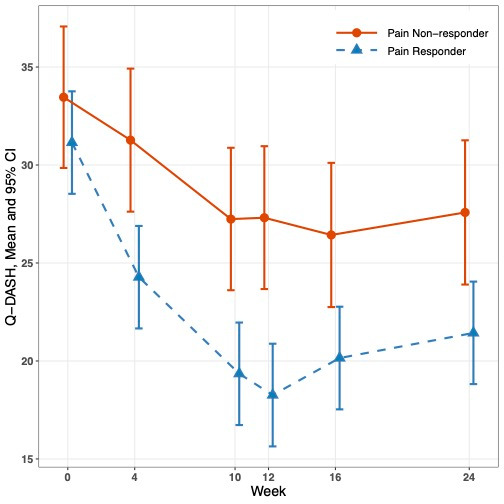

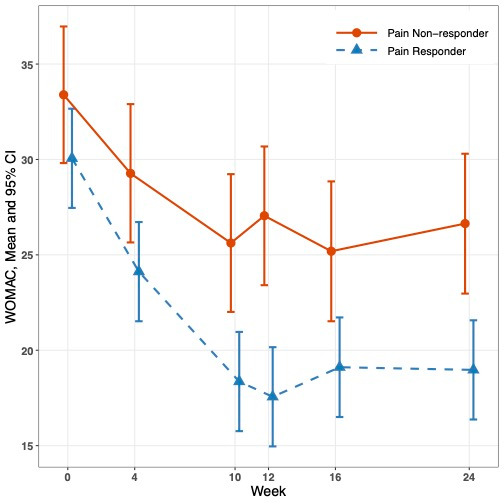
Q-DASH and WOMAC scores between pain responders and non-responders over 24 weeks.

Within the two acupuncture groups, there were 173 pain responders and 92 pain non-responders. Compared with pain non-responders, pain responders had a significantly greater reduction in average Q-DASH score by 6.74 points (95% CI 3.86, 9.62, *p* < 0.001) and in average WOMAC score by 6.16 points (95% CI 3.03, 9.29, *p* < 0.001) from baseline to week 12 (Figure 2 and Table 3).

## 4. Discussion

Functional limitations significantly impact the quality of life in patients with cancer experiencing chronic pain. In this secondary analysis of the PEACE trial, both electro-acupuncture and auricular acupuncture demonstrated efficacy in improving upper and lower extremity functional performance compared to a waitlist control. For lower extremity function, the benefits of both acupuncture interventions persisted up to 14 weeks post-intervention, and the reduction met the minimum clinically important difference for WOMAC function (MCID, 11 points) [26]. Although upper extremity function appeared to worsen over the follow-up period, it was still improved from baseline. However, the reduction did not meet the existing MCID (12–15 points) [27]. Auricular acupuncture demonstrated no significant difference from electro-acupuncture in facilitating pain-related functional rehabilitation. Additionally, patients who responded to acupuncture treatment (electro-acupuncture and auricular acupuncture) for pain relief experienced greater improvements in extremity function, highlighting the potential role of acupuncture in enhancing mobility and quality of life for patients with cancer with chronic pain.

Our study provides additional evidence that electro-acupuncture significantly improves chronic pain-related functional limitations in both upper and lower extremities, expanding the current understanding of its therapeutic benefits. Previous research has demonstrated acupuncture’s effectiveness in improving functional limitations in the post-surgical setting, such as in patients after neck surgery and in patients with breast cancer following surgery [28,29]. However, our research is the first to establish the benefits of electro-acupuncture for upper extremity function in a broader population experiencing chronic pain. For lower limb function, a systematic review found that 6–8 weeks of acupuncture intervention significantly reduced WOMAC pain subscale scores in breast cancer patients with aromatase inhibitor-induced arthralgia [30], though no significant improvements were observed in the WOMAC function subscale. Another RCT reported that acupuncture reduced trouble walking scores in patients with cancer with chemotherapy-induced peripheral neuropathy (CIPN), but functional performance was not objectively assessed using standardized instruments [31]. To the best of our knowledge, our study is the first to demonstrate that electro-acupuncture significantly improves WOMAC function scores in patients with cancer with chronic pain. These findings not only reinforce the role of electro-acupuncture as an effective intervention for functional impairment but also highlight its potential for broader clinical applications in pain management.

Auricular acupuncture is an emerging technique that has demonstrated effectiveness in pain reduction for patients with cancer. However, evidence remains limited regarding its impact on pain-related functional limitations. A systematic review suggested that auricular acupuncture may improve the quality of life in cancer patients experiencing pain [32]. Additionally, a pilot RCT among breast cancer patients found that auricular point acupressure reduced both pain intensity and pain interference with daily activities by 61% compared to a sham group [33]. Our study builds on this emerging evidence, demonstrating that auricular acupuncture significantly enhances extremity function with a similar effect as electro-acupuncture, which can be a viable and more convenient intervention for improving functional performance in patients with cancer with chronic pain.

Our results have certain implications for future clinical practice, especially for cancer-related pain rehabilitation. In both acupuncture groups, patients who exhibited a greater pain response to treatment also experienced greater improvements in functional performance. Conventional cancer rehabilitation and physical therapy typically focus on active and passive movements to enhance function; however, these interventions can sometimes exacerbate pain or discomfort, leading to reduced patient adherence and diminished effectiveness [34,35]. Our findings suggest that optimizing pain management with acupuncture prior to initiating rehabilitation may help patients better engage in and benefit from functional recovery programs. Supporting this approach, an RCT in patients with carpal tunnel syndrome demonstrated that combining acupuncture with physiotherapy resulted in superior pain relief and Q-DASH functional improvement compared to physiotherapy alone [36]. These results highlight the potential for an integrative, collaborative model where acupuncture is incorporated into conventional rehabilitation strategies to enhance pain management and functional outcomes for cancer patients.

The mechanisms underlying acupuncture’s effects on pain relief and functional improvement are not yet fully understood. Current research suggests that persistent nociceptor stimulation in chronic pain conditions lowers activation thresholds, leading to exaggerated responses to stimuli and contributing to functional impairments [37]. Additionally, chronic pain is associated with increased central nervous system excitability and heightened inflammatory responses, both of which can further limit physical function [38]. Psychological distress related to pain may also restrict daily activities and impair overall functional performance [39]. Acupuncture is believed to modulate pain perception by stimulating the release of endogenous opioids, serotonin, and norepinephrine, which can alleviate discomfort and improve mobility [40]. Furthermore, acupuncture has been shown to enhance microcirculation and reduce the release of pro-inflammatory cytokines, potentially aiding in functional recovery [41]. Notably, findings from our original PEACE trial demonstrated that both electro-acupuncture and auricular acupuncture improved mental health scores among cancer patients with chronic pain [21]. While acupuncture has demonstrated potential in both pain management and functional restoration, further research is needed to elucidate its precise mechanisms of action.

This study has several limitations. First, as this study involved secondary analyses of data from a prior clinical trial, its findings are intended to be exploratory in nature and should not be interpreted as definitive evidence. Second, since the primary trial was designed to assess pain management rather than functional performance, there was considerable variability in patients’ Q-DASH and WOMAC scores, with many patients experiencing minimal impairment. Given the absence of established cut-off values for these questionnaires, we included all participants in our analysis. It is possible that a more pronounced effect size would be observed if the study focused specifically on patients with greater functional limitations. Third, because the PEACE trial adopted a pragmatic design and did not include a sham control, the observed differences between acupuncture and waitlist groups represent overall treatment effectiveness rather than the specific physiological effects of needling. Finally, as the study was conducted in urban and suburban areas within an academic medical center, the generalizability of these findings to other settings—such as rural regions or community-based practices—may be limited.

In conclusion, among cancer survivors with chronic musculoskeletal pain, both electro-acupuncture and auricular acupuncture were associated with significant improvements in extremity functional performance compared to the waitlist control. Additionally, patients who exhibited a better pain response to acupuncture experienced greater functional gains, highlighting the potential link between effective pain management and improved mobility. These findings suggest that acupuncture may play a valuable role in addressing pain-related functional limitations in cancer survivors. Future research is warranted to further explore the integration of acupuncture into cancer rehabilitation and its long-term benefits for patients with pain-related functional impairments.

## Figures and Tables

**Table 1:** Baseline participant characteristics.

		No. (%)	No. (%)	No. (%)	No. (%)
Characteristics/Group		Overall N = 360	Electro-Acupuncture N = 145	Auricular Acupuncture N = 143	Waitlist Control N = 72
Age	Mean (SD), y	62.1 (12.7)	61.9 (13.2)	62.6 (11.3)	61.5 (14.4)
gender					
	Male	109 (30.3)	43 (29.7)	49 (34.3)	17 (23.6)
	Female	251 (69.7)	102 (70/3)	94 (65.7)	55 (76.4)
Race					
	Non-white	88 (24.4)	42 (29.0)	34 (23.8)	12 (16.7)
	White	272 (75.6)	103 (71.0)	109 (76.2)	60 (83.3)
Ethnicity					
	Hispanic	36 (10.1)	19 (13.2)	12 (8.5)	5 (7.0)
	Not Hispanic	321 (89.9)	125 (86.8)	130 (91.5)	66 (93.0)
Cancer type					
	Breast	165 (45.8)	66 (45.5)	67 (46.9)	32 (44.4)
	Prostate	41 (11.4)	16 (11.0)	18 (12.6)	7 (9.7)
	Colorectal/GI	15 (4.2)	5 (3.4)	4 (2.8)	6 (8.3)
	Lymphoma	51 (14.2)	22 (15.2)	19 (13.3)	10 (13.9)
	Melanoma	18 (5.0)	5 (3.4)	8 (5.6)	5 (6.9)
	Lung	12 (3.3)	5 (3.4)	7 (4.9)	0 (0)
	Other	58 (16.1)	26 (17.9)	20 (14.0)	12 (16.7)
Time since cancer diagnosis, mean (SD), y		6.2 (6.7)	6.1 (6.5)	6.1 (6.8)	6.5 (7.0)
Pain duration, mean (SD), y		5.3 (6.5)	5.7 (6.7)	4.8 (6.3)	5.5 (6.4)
Baseline measures					
Q-DASH, mean (SD) (0–100)		33.2 (19.8)	32.6 (19.9)	33.4 (19.3)	34.0 (20.9)
WOMAC-function, mean (SD) (0–100)		33.3 (20.3)	33.8 (20.7)	30.6 (19.4)	37.7 (20.3)

Abbreviations: y: years; SD: standard deviation; Q-DASH: Quick-Disability Arm/Shoulder/Hand; WOMAC: Western Ontario and McMaster Universities Osteoarthritis.

**Table 2:** Q-Dash and WOMAC across all treatment groups.

	Mean (95% CI)					
	Waitlist Control	Electro-Acupuncture		Auricular Acupuncture		Difference in Change from Baseline, Electro-Acupuncture to Auricular Acupuncture
Functional Performance	Baseline and Change from Baseline *	Baseline and Change from Baseline *	Difference from Waitlist Control in Change from Baseline	Baseline and Change from Baseline *	Difference from Waitlist Control in Change from Baseline	
Q-DASH						
Baseline	33.00 (31.08, 34.91)	33.00 (31.08, 34.91)	NA	33.00 (31.08, 34.91)	NA	NA
Week 4	−1.21 (−3.83, 1.40)	−4.95 (−6.80, −3.10)	−3.74 (−6.90, −0.58) *p* = 0.02	−6.02 (−7.95, −4.08)	−4.80 (−8.01, −1.60) *p* = 0.003	1.06 (−1.56, 3.68) *p* = 0.43
Week 10	1.66 (−1.00, 4.32)	−9.42 (−11.29, −7.55)	−11.07 (−14.29, −7.86) *p* < 0.001	−10.85 (−12.79, −8.92)	−12.51 (−15.76, −9.26) *p* < 0.001	1.43 (−1.21, 4.08) *p* = 0.29
Week 12	−2.65 (−5.31, 0.01)	−9.82 (−11.71, −7.94)	−7.18 (−10.39, −3.96) *p* < 0.001	−12.29 (−14.22, −10.36)	−9.64 (−12.89, −6.40) *p* < 0.001	2.47 (−0.18, 5.11) *p* = 0.07
Week 16	NA	−9.45 (−11.34, −7.56)	NA	−10.04 (−12.01, −8.06)	NA	0.59 (−2.09, 3.26) *p* = 0.67
Week 24	NA	−7.46 (−9.34, −5.57)	NA	−9.89 (−11.87, −7.92)	NA	2.44 (−0.24, 5.12) *p* = 0.07
WOMAC						
Baseline	33.13 (31.22, 35.04)	33.13 (31.22, 35.04)	NA	33.13(31.22,35.04)	NA	NA
Week 4	−0.65 (−3.46, 2.16)	−5.47 (−7.45, −3.49)	−4.82 (−8.21, −1.44) *p* = 0.005	−6.10 (−8.16, −4.04)	−5.45 (−8.88, −2.03) *p*=0.002	0.63 (−2.16, 3.42) *p* = 0.66
Week 10	−1.59 (−4.45, 1.27)	−11.10 (−13.11, −9.08)	−9.50 (−12.95, −6.06) *p* < 0.001	−10.36 (−12.44, −8.29)	−8.77 (−12.25, −5.29) *p* < 0.001	−0.73 (−3.56, 2.09) *p* = 0.61
Week 12	−3.84 (−6.70, −0.98)	−10.73 (−12.75, −8.71)	−6.89 (−10.33, −3.44) *p* < 0.001	−11.45 (−13.52, −9.39)	−7.61 (−11.08, −4.14) *p* < 0.001	0.72 (−2.10, 3.55) *p* = 0.61
Week 16	NA	−10.77 (-12.79, −8.75)	NA	−9.87 (−11.99, −7.76)	NA	−0.90 (−3.76, 1.97) *p* = 0.54
Week 24	NA	−10.32 (−12.34, −8.31)	NA	−9.78 (−11.88, −7.76)	NA	−0.55 (−3.40, 2.30) *p* = 0.71

* For Baseline/Week 0, this column contains the baseline mean and CI, and for Week 4, 10, 12, 16, and 24, this column contains the mean change from baseline. Abbreviations: CI: confidence interval.

**Table 3:** Q-DASH and WOMAC results across all pain responders and non-responders.

Functional Performance	Mean (95% CI)		
	Pain Responder N = 173	Pain Non-Responders N = 92	Difference in Change from Baseline, Pain Responders to Non-Responders
	Baseline and Change from Baseline *	Baseline and Change from Baseline *	
Q-DASH			
Baseline	31.16 (28.55, 33.77)	33.40 (29.80, 37.00)	NA
Week 4	−6.87 (−8.53, −5.21)	−2.18 (−4.53, 0.16)	−4.68 (−7.56, −1.81) *p* = 0.0014
Week 10	−11.80 (−13.46, −10.14)	−6.21 (−8.54, −3.88)	−5.59 (−8.44, −2.73) *p* = 0.0001
Week 12	−12.88 (−14.55, −11.22)	−6.14 (−8.49, −3.80)	−6.74 (−9.62, −3.86) *p* < 0.001
Week 16	−10.99 (−12.66, −9.33)	−7.03 (−9.42, −4.63)	−3.97 (-6.89, −1.05) *p* = 0.0077
Week 24	−9.71 (−11.38, −8.05)	−5.87 (−8.27, −3.47)	−3.84 (−7.76, −0.92) *p* = 0.01
WOMAC			
Baseline	30.06 (27.46, 32.66)	33.39 (29.81, 36.97)	NA
Week 4	−5.94 (−7.74, −4.14)	−4.11 (−6.65, −1.57)	−1.83 (−4.94, 1.29) *p* = 0.25
Week 10	−11.70 (−13.51, −9.89)	−7.77 (−10.29, −5.25)	−3.93 (−7.03, −0.83) *p* = 0.013
Week 12	−12.50 (−14.31, −10.69)	−6.34 (−8.89, 3.79)	−6.16 (−9.29, −3.03) *p* = 0.0001
Week 16	−10.95 (−12.77, −9.13)	−8.20 (−10.80, −5.60)	−2.75 (−5.92, 0.43) *p* = 0.09
Week 24	−11.09 (−12.91, −9.28)	−6.75 (−9.35, −4.15)	−4.34 (−7.51, −1.17) *p* = 0.0073

* For Baseline/Week 0, this column contains the baseline mean and CI, and for weeks 4, 10, 12, 16, and 24, this column contains the mean change from baseline.

## Data Availability

The data are available from the corresponding author upon reasonable request.
